# Prepuce: Phimosis, Paraphimosis, and Circumcision

**DOI:** 10.1100/tsw.2011.31

**Published:** 2011-02-03

**Authors:** Yutaro Hayashi, Yoshiyuki Kojima, Kentaro Mizuno, Kenjiro Kohri

**Affiliations:** Department of Nephro-urology, Nagoya City University Graduate School of Medical Sciences, Japan

**Keywords:** prepuce, phimosis, paraphimosis, circumcision, preputioplasty

## Abstract

Phimosis is a condition in which the prepuce cannot be retracted over the glans penis. Actually, physiologic phimosis is common in male patients up to 3 years of age, but often extends into older age groups. Balanoposthitisis a common inflammation occurring in 4–11% of uncircumcised boys. Circumcision is generally undertaken for three reasons: first, as an item of religious practice, typically neonatally although occasionally transpubertally, as a rite of passage; second, as a prophylactic measure against future ailments for the reduction in the risk of penile cancer, urinary tract infection, and sexually transmitted infection; and third, for immediate medical indication. Balanitisxeroticaobliterans is an infiltrative skin condition that causes a pathological phimosis and has been considered to be the only absolute indication for circumcision. Various kinds of effective alternatives to circumcision have been described, including manual retraction therapy, topical steroid therapy, and several variations of preputioplasty. All of these treatments have the ability to retract the foreskin as their goal and do not involve the removal of the entire foreskin. Paraphimosis is a condition in which the foreskin is left retracted. When manipulation is not effective, a dorsal slit should be done, which is usually followed by circumcision.

## PREPUCE

### Natural History of Prepuce

At birth, there is normally a physiologic phimosis or inability to retract the foreskin because natural adhesions exist between the prepuce and the glans. During the first 3–4 years of life, as the penis grows, epithelial debris (smegma) accumulates under the prepuce, gradually separating the foreskin from the glans. Intermittent penile erections cause the foreskin to become completely retractable.

Gardiner reported that at birth, <5% of boys have a fully retractable prepuce and this figure increases to 15% at 6 months, 50% at 1 year, 80% at 2 years, and approximately 90% at 3 years in the U.K.[1]. Oster recorded more than 9,000 observations in Danish boys and demonstrated that 90% of foreskins can be retracted by 3 years of age and <1% of males have phimosis by 17 years of age[2]. In 1996, Kayaba et al. evaluated preputialretractability in 603 Japanese boys 0–15 years of age and reported that the incidence of completely retractable prepuce increased 0% at age 6 months to 62.9% by 11–15 years of age, while the incidence of completely unretractable prepuce decreased 47.1% at age 6 months to 0% by 11–15 years of age[3]. In 1997, Imamura investigated the condition of Japanese prepuce in 3,238 infants and in 1,283 children aged 3 years[4]. The incidence of completely retractable prepuce increased from 3.0% in infants aged 1–3 months to 38.4% in children aged 3 years, while the incidence of completely unretractable prepuce decreased from 88.5% in infants aged 1–3 months to 35.0% in children aged 3 years. In 2006, Hsieh et al. examined the foreskin and external genitalia of 2,149 Taiwanese schoolboys and showed that 50% of 7-year-old boys had phimosis, which decreased to 8% at age 13 years[5]. In 2009, Yang et al. investigated preputialretractability in 10,421 Chinese boys aged 0–18 years and reported that the rate of phimosis decreased with age from 99.7% at birth to 6.81% in adolescence[6].

## Problems of Prepuce

### *Phimosis*

Phimosis is a condition in which the prepuce cannot be retracted over the glans penis. It could be further defined as physiologic, as in infancy and childhood, or pathologic. Pathologic phimosis would result from inflammatory or traumatic injury to the prepuce resulting in an acquired inelastic scar that prevents retraction. Forceful disruption of physiologic adhesions in infants no doubt encourages pathologic phimosis. Physiologic phimosis is common in male patients up to 3 years of age, but often extends into older age groups[1,2,3].

### *Balanoposthitis*

Balanitis is the term for inflammation of the glans penis. Posthitis is defined as inflammation of the prepuce. Balanoposthitisis inflammation of both (Fig. 1). It is fairly common, occurring in 4–11% of uncircumcised boys[7,8,9]. The etiology is unclear and no cause can be identified in many cases, although infection, mechanical trauma, contact irritation, and contact allergy are cited[10].

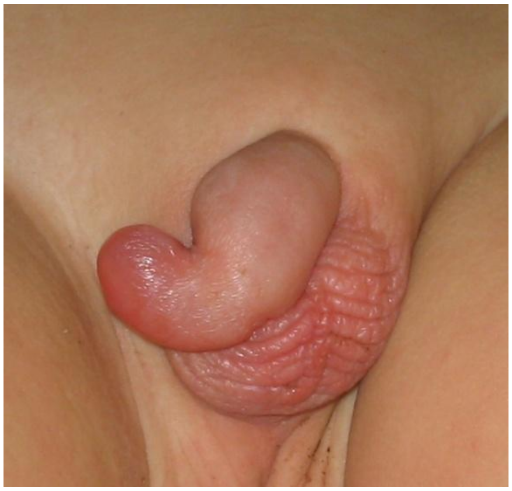

**FIGURE 1.**Balanoposthitis.

Management of balanoposthitis includes improved hygiene with gentle foreskin retraction, sitz baths, and cleaning of the foreskin[11]. Topical ointment and oral antibiotics are indicated for suspected acute bacterial balanoposthitis. Because Group A beta hemolytic streptococcus is a common bacterial cause of balanoposthitis, first-generation cephalosporins or penicillins have been typically recommended[12,13].

## Paraphimosis

Paraphimosis is a condition in which the foreskin is left retracted because of entrapment of the tight prepuce proximal to the corona (Fig.2). The glans engorges and the prepuce becomes edematous because of lymphatic and venous congestion. This could happen because boys have been encouraged to retract the foreskin for physiological phimosis by parents or medical staff. In most instances, manual compression can reduce the preputial edema within the first few hours. In difficult cases, various techniques are described, including applying granulated sugar to the penis[14], adding multiple punctures to the edematous foreskin before compression[15], injecting hyaluronidase beneath the narrow band to release it, and wrapping the distal penis in a saline solution–soaked gauze swab and squeezing gently but firmly for 5–10 min[16]. Thereafter, physicians are supposed to push forcefully on the glans with the thumbs, while pulling the foreskin with the fingers. However, an emergency dorsal slit may be necessary in late cases. Some authors advise circumcision for paraphimosis because of its tendency to recur, whereas others insist that circumcision is not mandatory because the foreskin will continue to develop normally.

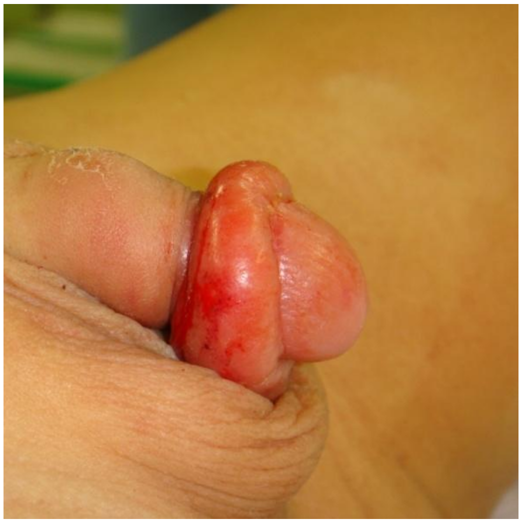

**FIGURE 2.**Paraphimosis.

## BalanitisXeroticaObliterans

Balanitisxeroticaobliterans (BXO), known as lichen sclerosus, is an infiltrative skin condition that causes a true phimosis and a clinically recognizable lesion at the tip of the prepuce[17]. It is usually distinguished by a ring of hardened tissue with extensive scarring, a whitish color at the tip of the foreskin, and edema (Fig. 3). It has been said to be a common underlying cause of persistent nonretractability of the foreskin at puberty[2], while the overt lesion is rarely seen in children under 5 years of age[18]. However, Meuli et al. found BXO in 15% of children undergoing circumcision for phimosis[19]. Presentation is with inability to retract the prepuce, discomfort after micturition, and occasional minor obstructive signs[20].

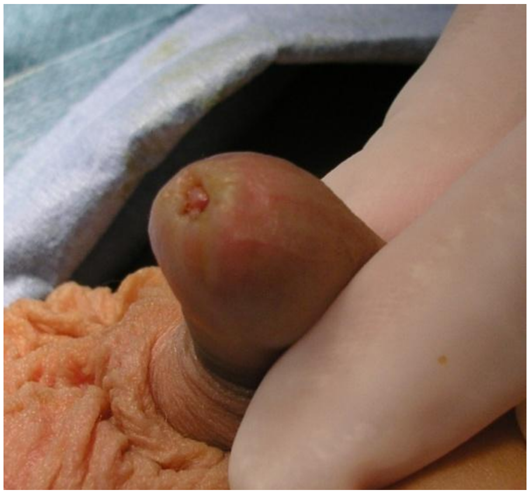

**FIGURE 3**.Balanitisxeroticaobliterans.

Definitive diagnosis was reached with biopsy, which showed hyperkeratosis with follicular plugging, atrophy of the stratum spinosumMalpighii with hydropic degeneration of basal cells, lymphedema, hyalinosis and homogenization of collagen in the upper dermis,and inflammatory infiltration in the mid dermis.

Whether or not the BXO lesion will respond to the topical steroid is still unclear, although administration of topical steroid has been proven to be effective in approximately 80% of physiological phimosis. Vincent and Mackinnon evaluated 56 boys with clinical features of BXO and reportedthat 17.9% of patients showed complete resolution after treatment with topical steroid after 3 months, which increased to 30.4% after an average of 14 months of the treatment[21]. Kiss et al.performed histological evaluation of topical steroid application for BXO, and concluded that steroid treatment tends to be effective when the inflammatory mechanism is active and irreversible tissue damage has not occurred, including cases of the early and intermediate histological forms of the disease, whereas in the late disease type in which irreversible changes are exemplified by severe degeneration and atrophy of the genital skin, treatment is ineffective or at best only slows further worsening[22].

The preferred treatment for BXO has been circumcision, which will remove all the affected tissue. Preputioplasty is not an option because the continuing inflammatory process results in recurrent stenosis of the preputial orifice. Remaining lesions on the glans nearly always regress or resolve following circumcision. Meatotomy or meatoplasty is needed in cases of severe meatal involvement, and postoperative application of topical steroid may lessen the risk of subsequent restenosis.

## CIRCUMCISION

### Circumcision as Religion and Rite of Passage

Globaly, most circumcisions are performed for religious reasons and are a fundamental part of the Jewish and Muslim faiths.

In the Jewish belief, circumcision is a covenant between God and Abraham, as written in the Bible(Genesis) and should be performed by a mohel when the boy is 8 days old[23].

Muslim society considers it a tradition of the Prophet Mohammed (Sunnah) to introduce the boy into the religious Islamic community, although it is not mentioned in the Holy Koran[24].

Male circumcision that is performed for any reason other than physical, clinical need is termed nontherapeutic (or sometimes ritual) circumcision.

## Circumcision as Prophylactic Therapy

### *Prophylactic Circumcision to Prevent Urinary Tract Infection (UTI)*

Incomplete preputial separation has been considered responsible for colonization of the prepuce by pathogens, which leads to balanoposthitis or UTI.

The incidence of UTIhas been reported to be lower in circumcised than uncircumcised infants[25,26]. An unretractable prepuce has been considered responsible for colonization of the prepuce by pathogens, which leads to UTI. An association between a decreased incidence of UTIand circumcision during the first year of life has been reported[27].

Hiraoka et al. analyzed 100 consecutive children (males and females) who developed febrile UTI, and investigated preputialretractability in 64 boys with febrile UTIand 714 healthy boys who underwent public health checkups[28]. The external urethral meatus could not be observed by gentle retraction in 85% of the boys under the age of 7 months with their first urinary infection,compared to 42% of healthyboys. They mentioned that the tightly covered urethral meatus may cause urinary infection frequently found in uncircumcised young infants, although spontaneous resolution of phimosis could be expected with maturation of prepuce.

### *Prophylactic Circumcision to Prevent SexuallyTransmitted Infection(STI)*

Whether early childhood circumcision reduces the risk of future STIs other than human immunodeficiency virus (HIV) has been controversial.

In 1998, Moses et al. analyzed 11 studies and reported a strong association between the lack of circumcision and chancroid, syphilis, genital herpes, and gonorrhea, but no association with urethritis other than gonorrhea and genital warts[29].

Cook et al. performed a cross-sectional study of 2,776 heterosexual men attending an STI clinic in 1998 and identified a positive relationship between lack of circumcision and both syphilis and gonorrhea[30].They recognized a negative relationship with genital warts and no relationship with genital herpes or nongonococcal urethritis.

Laumann et al. conducted a study of 1,410 American men aged 18–59 years, examining the prevalence of circumcision across various social groups, and the health and sexual outcomes of circumcision[31]. They did not find a statistically significant difference in the incidence of STIs based on circumcision status.

Weiss et al. conducted a systematic review and meta-analysis for male circumcision using 26 articles in 2006[32]. They reported that circumcised men are at low risk of chancroid and syphilis, but there is less association with herpes simplex type 2.

### *Prophylactic Circumcision to PreventHuman Immunodeficiency Virus (HIV)*

Three randomized controlled trials (RCTs) in Africa confirmed that male circumcision reduces the rate of female-to-male HIV transmission by 55–76%[33,34,35]. These three RCTs were abandoned before their designed completion because of significant reductions in HIV incidence in the circumcised groups. Lack of circumcision has long been recognized as a risk factor for HIV acquisition in heterosexual men[29]. This makes biological sense because superficial Langerhans cells, which express HIV-1 receptors, are more prevalent in the male foreskin than in the remainder of the penis[36]. Additionally, decreased keratinization of the foreskin increases susceptibility to minor trauma during intercourse, potentially aiding the passage of HIV[37].

In 2009, an expert group, consisting of the Joint United Nations Programme on HIV/AIDS (UNAIDS), the World Health Organization (WHO), and the South African Centre for Epidemiological Modelling and Analysis (SACEMA), reviewed six mathematical models using the latest data on the effect of circumcision on HIV prevention[38]. The models predicted that one new HIV infection would be averted for every 5–15 men newly circumcised.

### *Prophylactic Circumcision to Prevent Penile Cancer*

Incidence of carcinoma of the penis varies greatly with the standards of hygiene, and the religious and cultural practices of different countries. It is usually seen in men who have poor standards of penile hygiene.

In men who have not been circumcised, tumor development has been attributed to the chronic irritative effects of smegma, a by-product of bacterial action on desquamated cells within the preputial sac. Closed preputial sacs promote development of penile carcinoma by a carcinogen[39]. Smegma has been implicated as the carcinogenic agent and, although definite evidence has not been established, its association with the development of penile cancer has been widely observed.

Phimosis is present in 25–75% of patients with penile carcinoma[40,41,42,43].The true incidence of phimosis might be even higher, since information about its presence is not available in many patients in whom the prepuce has been destroyed by the tumor.

Circumcision protects against penile carcinoma and is a well-established prophylactic measure that almost eliminates the occurrence of penile cancer. Neonatal circumcision is done for all Jewish males and carcinoma of the penis is almost nonexistent in this population[40]. Neonatal circumcision reduces the risk of penile cancer by at least ten times[29].In Muslims, who circumcise boys before puberty, penile carcinoma is rare[44].

Adult circumcision does not offer protection from subsequent development of disease, suggesting that some period of exposure to smegma might be necessary for carcinogenesis, and accounts for the decreased effectiveness of pubertal circumcision and the negligible protective effect of adult circumcision[45].

In developed countries, penile cancer is a relatively rare disease, with an incidence of <1 in 100,000[46]. The absence of RCT evidence, combined with the rarity of penile cancer, suggests that circumcision is not justified for the sole purpose of protecting against penile cancer.

## METHODS OF CIRCUMCISION

Neonatal circumcision is most commonly performed under local anesthesia outside the operating room, using one of three techniques. Two of these, the Gomco clamp and the Plastibell device, require the use of a specialized apparatus. The Mogen clamp is a simple instrument used in conjunction with conventional sharp and blunt dissection. In the beginning of the operation, all three techniques assure inspection of the glans and mobilization of the foreskin from physiological adhesions. Both the Mogen and the Gomco clamps protect the glans, while producing crush injury to the prepuce, which is then surgically removed. Jewish ritual circumcisions are usually conducted with a Mogen clamp[47,48]. The Plastibell device induces necrotic tissue, which is sloughed off, along with the plastic shield, within 1–2 weeks. In the meantime, the infant voids through the open end of the bell. A retrospective cohort study of 5,521 American boys compared neonatal circumcision results using the Gomco clamp and the Plastibell device. The overall complication rate of 0.2% did not differ between the two groups. However, the Plastibelldevice was associated with more infections, whereas the Gomcoclamp was associated with more dehiscence and removal of too much skin[49].

In older children, circumcision is usually performed in the operating room under general anesthesia, with the addition of a local anesthetic penile or caudal block to provide good analgesia during and immediately after the surgery. Because circumcision devices seem to be less adequate for older children, most circumcisions are conducted by the sleeve or freehand technique.

Any remaining adhesions between the glans and prepuce are bluntly lysed. A circumferential incision is made overlying the coronal impression of the glans through the skin. On the ventral surface, the skin incision should be in a Vshape opposite the frenulum. The foreskin is then retracted and a circumferential incision is made in the inner prepuce approximately 0.5 cm proximal to the coronal sulcus, preserving the frenular arteries. After both incisions are made, a “sleeve” of preputial tissue is created. On the dorsal surface, a pair of scissors is used to create a plane superficial to Buck’s fascia between the two circumferential incisions. The ring of the prepuce is incised along this plane and then removed. The skin and the inner prepuce are reapproximated with absorbable sutures after hemostasis is achieved.

When the prepuce cannot be retracted easily, a dorsal slit is initially required from the tip of the prepuce extending to the circumferential outer skin incision and proximal to the coronal sulcus at the inner side. The edges of the preputial incision are grasped and both layers of the prepuce are divided circumferentially on the marked line, leaving the frenulum in place. After the removal of the prepuce, the remaining steps are the same as the sleeve technique.

In circumcision, a carbon dioxide laser beam can be used as a cutting device, which significantly decreases the incidence of postoperative bleeding and also postoperative edema[50,51,52].

Although an absorbable suture of a fine caliber provides a good cosmetic result, a tissue glue supplied with a fine nozzle applicator gives a cosmetically pleasing result[53,54,55].

## ALTERNATIVE TREATMENTS TO CIRCUMCISION

### Manual Retraction Therapy

Cooper et al. performed foreskin retraction on 106 boys under general anesthesia. Of them, 93 had presented with symptoms (balanoposthitis, dysuria, preputial ballooning, or retained smegma) and 13 had been referred because of a nonretractable foreskin alone. After the therapeutic retraction under anesthesia, the parents were asked to retract their child’s foreskin constantly. As a result, the symptoms resolved in 85% of the boys and the foreskin became fully retractable in 62%[56].

Tsugaya et al. practiced the manual retraction therapy for 98 boys between the ages of 1 month and 12 years. They reported that exposure of the glans by forceful retraction should not be performed because readhesion between the glans and prepuce occurred in 68% of those who underwent the retractiontherapy[57].

Rickwood et al. mentioned that BXO commonly occurs as a result of forceful retraction of the prepuce[18]. Iatrogenic injury from forcible retraction of the prepuce is a common cause of true phimosis. Preputial injury leads to cicatrix formation, which narrows the preputial aperture.

### Topical Steroid Therapy

Since 1993, various kinds of topical ointment have been successfully used to treat phimosis in boys. Success rates ranging between 67 and 95% have been reported with topical application ofbetamethazonecream as a highly potent steroid[58,59,60]. Several studies have noted success with the application of clobetasol propionate as an ultrahigh potent steroid and described success rates between 70 and 92%[61,62,63]. As a medium potent steroid, monometasonefuroate topical cream has had success rates of 66[64] and 88%[65], and triamucinoloneacetonide topical cream has had success rates of 76[66] and 84%[67]. In addition, even with a low potent steroid, hydrocortisone, two studies showed satisfactory success rates of 86[68] and 90%[69]. The anti-inflammatory and immunosuppressive effects[70], as well as the skin thinning effects[71], of the topical steroids may explain their effectiveness. No adverse side effects were reported in all potent grades of these steroid treatments.

Pileggi et al. evaluated cortisol secretion in 31 boys by the measurement of salivary cortisol before starting treatment and after 8 weeks of topical treatment for phimosis with 0.05% clobetasolpropionate[72]. They mentioned that the salivary cortisol level should be considered as a laboratory marker in long-term treatment or during repeated cycles in order to detect possible hypothalamus-pituitary-adrenal axis suppression, although clobetasol propionate does not affect the axis in most patients.

On the other hand, Muller and Muller first applied topical conjugated equine estrogen to treat phimosis, expecting that it would increase collagen and water content of the skin tissue[73]. According to additional reports, the topical application of estrogen ointment for phimosis revealed successful results in 76–100%[74,75,76]. Although the response rate seemed to be consistent to that of the other topical steroid therapies, gynecomastia occurred during the treatment as a side effect in several reports. Yanagisawa et al. commented that prolonged application of estrogen ointment on a poorly responding foreskin may increase the patient’s risk to side effects[75].

## Dorsal Slit

The dorsal slit, a longitudinal incision of the dorsal preputial skin, is used to release the paraphimosis or severe balanitis with urinary retention when the prepuce cannot be retracted easily[77]. Emergent circumcision does not tend to be selected because it is technically difficult due to massive edema or it would carry a greater risk of postoperative infection.

However, in Japan, many physicians conductedthe dorsal slit for boys with phimosis even in nonemergent situations. Matsuoka et al. performed the dorsal slit for 43 children with phimosis and during the follow-up (from 1 to 12 years, mean: 4 years), they interviewed and examined 20 responders for the questionnaires about postoperative appearance[78].The results showed that 30% of them expressed some dissatisfaction with the penile appearance, and about half were apparently recognized to have abnormal appearance (ventral-sided redundant foreskin) compared with usual boys from the view of the operators.

## Preputioplasty

The dorsal slit, as traditionally and still occasionally performed, israrely to be recommended because the cosmetic result is unsatisfactory and, hence, formal circumcision almost always ensues. However, as mentioned before, in Japan, routine neonatal circumcision has never been conducted so far. Therefore, genital appearance with exposed glans after circumcision would look unusual among Japanese boys, even if surgical repair was eventually chosen as a prophylactic therapy for pediatric phimosis.

Preputioplasty, with preservation of the foreskin, has been proposed as an alternative to circumcision and may take the form either of a limited dorsal slit, with transverse suture[79], or longitudinal incision of the constricting ring proximal to the preputial meatus, again with transverse suture[80].

Dean et al. developed a geometric variant of the dorsal slit procedure, which added a ventral slit to achieve a natural appearance of intact foreskin and to be easily fully retractable[81].

Lane and South described a lateral preputioplasty as a variant of the dorsal slit[82]. In this procedure, two laterally placed longitudinal incisions were made and the defects were sutured transversely. They advocated that the lateral placement of the incisions provides cosmetic improvement over the dorsal approach, and avoids the impairment of frenular area over circumcision or other procedure including ventral slits.

A triple-incision preputioplasty was described by Welsh in 1936. The technique consisted of three longitudinal, full-thickness skin incisions across the stenotic ring down to the inner preputial layer and transversal suturing of the three defects to enlarge a phimotic ring(Fig. 4). Wåhlin modified the procedure and three rhomboid-shape defects made by the longitudinal incisions were closed with interrupted sutures placed obliquely in the middle of each incision[83].Fischer-Klein and Rauchenwald undertook the monitoring of the triple-incision preputioplasty modified by Wåhlin for 197 boys and were able to reassess 65% of parents and children[84]. They reported that 84% of parents were satisfied with the function and 80% answered a good cosmetic outcome. They mentioned that a slight rotation and shortening of the prepuce will be achieved, which is equally distributed around the whole circumference of the penis, avoiding the so-called “dog ears”preputialdeformity. They also commented that three parallel displaced suture lines are more likely to prevent restenosis than an almost continuous ring of transverse scars.

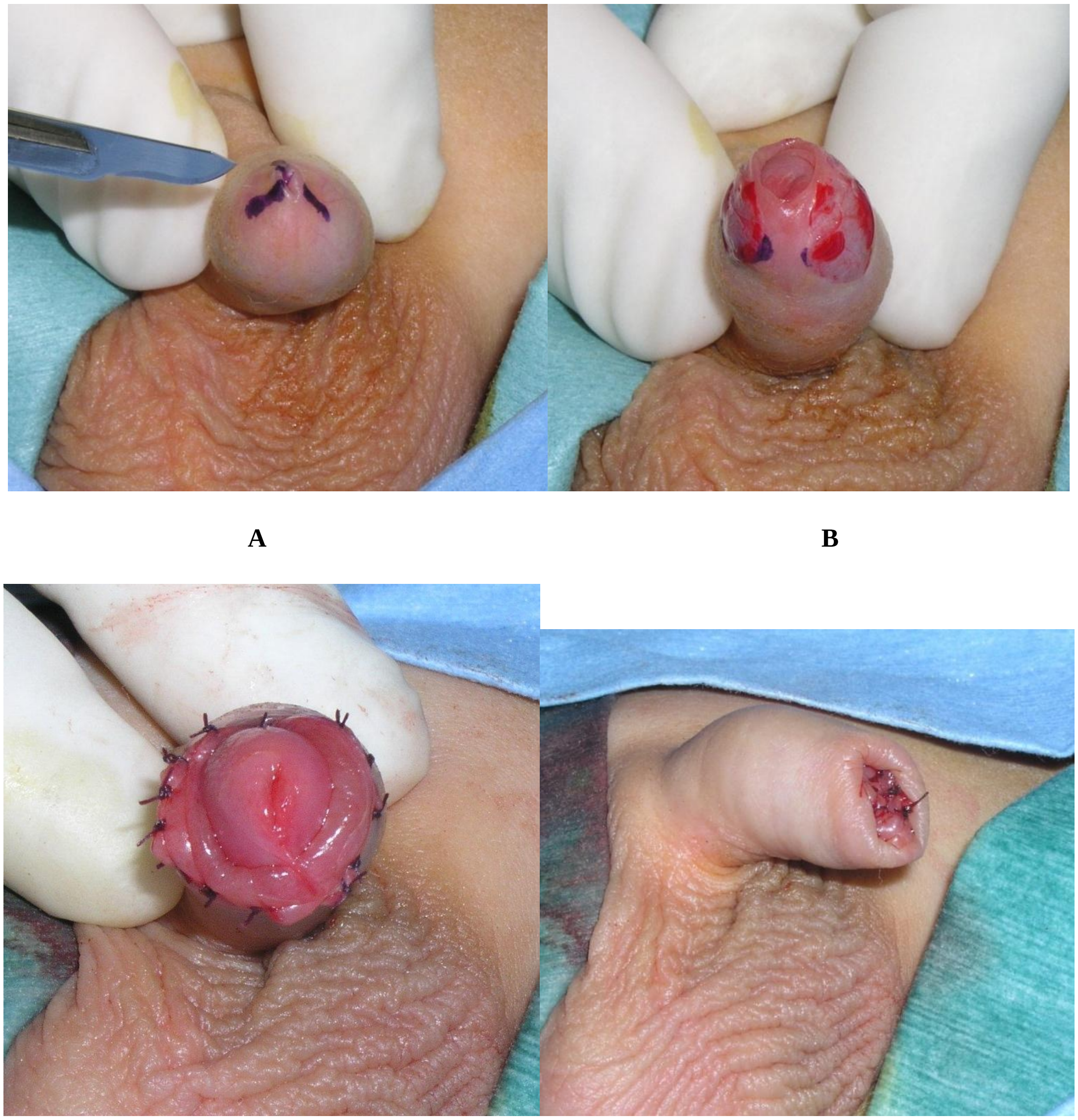

A B

C D

**FIGURE 4**.Triple-incision preputioplasty.

After the triple-incision preputioplasty, Pascotto and Giancotti added frenulotomy and two more incisions between the previous ones, which are left to heal spontaneously, and reported that the devised technique resulted in no recurrence, normal preputial anatomy, and function in all 22 children[85].

A Y-V plasty procedure(Fig. 5) to relieve phimosis was first described by Hoffman et al. in 1984[86].Multiple Y incisions across the narrow preputial ring were conducted. The tips of the flaps were advanced proximally and closed as multiple Vs, like a sawtooth.Forty-four patients underwent the procedure with an excellent result functionally and cosmetically. Nieuwenhuijs et al. carried out a retrospective study between 47 patients treated with a Y-V plasty and 18 treated by transversely closed longitudinal incisions of the narrow preputial ring, with recurrence rates of 4.3 and 11%[87].They concluded that a Y-V plasty could be an alternative to circumcision in the treatment of phimosisresistant to a topical steroid therapy.

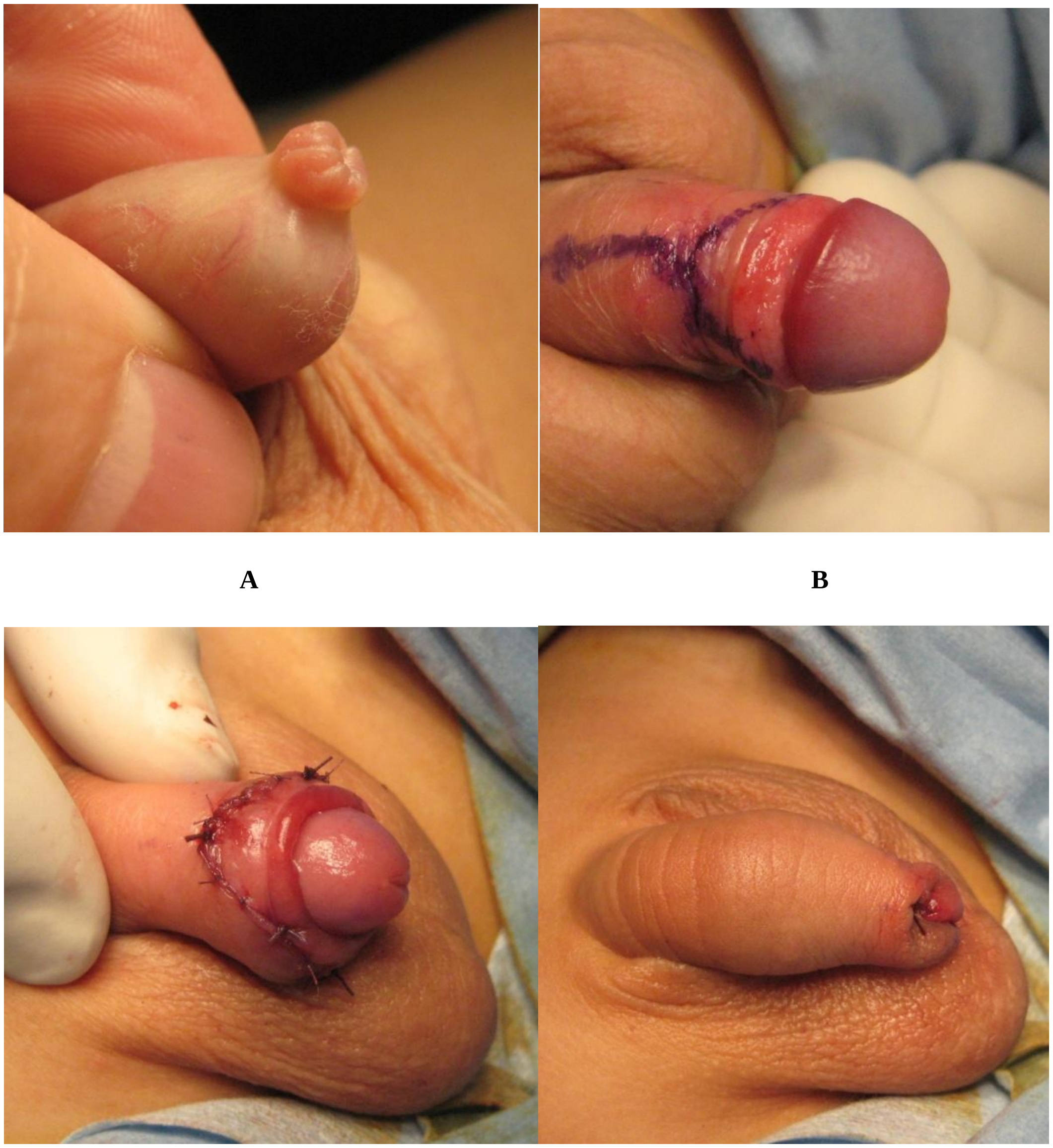

A B

C D

**FIGURE 5.**Y-V plasty.

Dessanti et al. developed a T-V plasty to enlarge the stenotic ring of prepuce by a transversal widening on the dorsal side[88]. Consequently, they achieved excellent cosmetic results in all 26 children at 7 days after operation and at 1-year follow-up.

Additional variations to these preputioplasties include a four V-flap repair[89], Z plasty[90], andhelicoidsplasty[91], and no complications were reported.
